# Mortality from contact-related epidemics among indigenous populations in Greater Amazonia

**DOI:** 10.1038/srep14032

**Published:** 2015-09-10

**Authors:** Robert S. Walker, Lisa Sattenspiel, Kim R. Hill

**Affiliations:** 1 Department of Anthropology, University of Missouri, Columbia MO, USA; 2 School of Human Evolution and Social Change; Institute of Human Origins, Arizona State University, Tempe AZ, USA

## Abstract

European expansion and contact with indigenous populations led to catastrophic depopulation primarily through the introduction of novel infectious diseases to which native peoples had limited exposure and immunity. In the Amazon Basin such contacts continue to occur with more than 50 isolated indigenous societies likely to make further contacts with the outside world in the near future. Ethnohistorical accounts are useful for quantifying trends in the severity and frequency of epidemics through time and may provide insight into the likely demographic consequences of future contacts. Here we compile information for 117 epidemics that affected 59 different indigenous societies in Greater Amazonia and caused over 11,000 deaths between 1875 and 2008, mostly (75%) from measles, influenza, and malaria. Results show that mortality rates from epidemics decline exponentially through time and, independently, with time since peaceful contact. The frequency of documented epidemics also decreases with time since contact. While previous work on virgin soil epidemics generally emphasizes the calamity of contacts, we focus instead on improvements through time. The prospects for better survivorship during future contacts are good provided modern health care procedures are implemented immediately.

European colonization of the New World brought catastrophic depopulation to indigenous societies^1^,^2^,^3^,^4^,^5^,^6^,^7^,^8^,^9^. Many of the same external threats of epidemics, violence, and displacement continue to adversely affect indigenous populations to this day^6^,^10^,^11^. Some of the smallest and most vulnerable human groups are the more than 50 isolated indigenous societies in Amazonia who have limited contact with the outside world^12^,^13^. Little scientific attention has been paid to the likely demographic outcomes of future contacts^14^, but such an endeavor is important given the precariousness of their situation^12^,^15^,^16^,^17^.

The introduction of novel infectious diseases was arguably the most devastating consequence of European colonization^8^,^9^,^18^. At the time of early colonization, many indigenous populations of the Americas had no prior exposure to pathogens that had become common in Europe. Such populations are often referred to as virgin soil populations^2^. In such populations, epidemics caused by acute infectious pathogens (called virgin soil epidemics) can be extremely severe, resulting in high mortality and complete social disruption. In small indigenous communities in Amazonia and elsewhere, this situation occurred not only at the time of initial European colonization, but repeatedly, albeit relatively rarely and widely dispersed in time over the succeeding centuries^4^,^5^,^6^.

The rarity of epidemics in isolated populations is well understood. In 1957, a statistician, Maurice Bartlett, examined several time series of measles epidemics from 19 towns in England and Wales and showed that there was a relationship between the mean time between epidemics and the size of a population^19^. Based on his results, he proposed 3 fundamental measles epidemic patterns. Type I waves occurred in large cities of over 250,000 residents and were characterized by constant, but small numbers of cases and occasional larger epidemic outbreaks. Type II waves occurred in populations between about 10,000 and 250,000 people, and consisted of discrete but regular epidemics which were separated by time spans where no measles cases were present. Type III waves were characteristic of small populations and consisted of sporadic epidemics whenever a disease was introduced but an inability to maintain a disease without exogenous introductions. The size of these populations was so small that outbreaks would spread quickly through the population, but the rapid depletion of susceptible individuals would break epidemic chains and result in local extinction of the disease^20^,^21^.

In a test of Bartlett’s ideas, Black^22^ gathered data on measles in 19 island populations that varied in size. He found a correlation between population size and the presence of regular measles epidemics, but noted that other factors, in particular population density and connections to the outside world, were also important. Cliff and Haggett^23^ expanded and updated Black’s study and came to the same general conclusions. What these studies indicate is that populations such as the majority of indigenous Amazonian tribes, which are both isolated and small in size, tend to experience epidemics that are dispersed in time and that tend to affect high proportions of the population because the rarity of outbreaks means that most individuals lack prior exposure to the pathogen and have no pre-existing immunity.

Although the rarity of epidemics is understood, there is little consensus concerning the underlying mechanisms behind how such immense depopulation occurred with contact. One view is that native immune systems are inefficient because of high genetic homozygosity. New World indigenous populations and especially Amazonians are characterized by low underlying genetic variation^24^,^25^,^26^,^27^ as well as different frequencies of alleles in immune system genes^28^. It is possible that these genetic characteristics may facilitate destructive epidemics of genetically-similar hosts^8^,^29^. South America is at the end of a long chain of serial founder populations and indigenous populations have low genetic diversity due to the serial founder effect and loss of alleles as populations migrate out through time. Heterozygous alleles at loci in the major histocompatibility complex are known to give better disease resistance once exposed to a certain class of pathogens^28^,^29^. A related genetic hypothesis is that differential selective pressures are key, that natural selection has not favored alleles because New World populations did not go through the same selective filters that Old World populations have and hence do not have the alleles that are most resistant to certain classes of pathogens^21^. The relatively small sizes of traditional small-scale forager and horticultural populations, mostly isolated from one another except through brief hostile interactions, are not conducive to long-term perpetuation of explosive epidemic diseases and hence did not exert strong selective pressure on effective immune defense against diseases that arose in large Old World populations^21^. Genetic traits and alleles in Amazonian groups (both indigenous and urban) have been studied to see if their variation is related to susceptibility for a variety of infectious diseases and pathogens including malaria^30^,^31^,^32^, tuberculosis^33^,^34^,^35^,^36^, *Helicobacter pylori*^37^,^38^, and leprosy^39^.

Even though there is considerable evidence of genetic differences between New World indigenous populations and other groups, mechanistic evidence that those differences underlie the increased severity of diseases in indigenous groups has remained elusive, although susceptibility to tuberculosis for those with alleles that are common in the Ache (Paraguay) is suggestive evidence^35^,^36^. Some studies suggest that many native Amazonians appear to mount normal immune responses to measles and vaccinations^20^,^40^,^41^,^42^,^43^. Furthermore, aside from a few traits that clearly alter mechanisms related to pathogenicity, studies linking specific genes to infectious diseases have not been able to adequately account for observed differences in susceptibility. What is needed to make the case for many of these genes is to go beyond simple associations indicated through correlation analyses and identify mechanisms that can explain the observed differences among individuals. Examples of such mechanistic evidence include explanations for the high frequency of the CCR5-∆32 deletion in European populations, which changes the structure of the CCR5 cytokine receptor and prevents HIV from entering a cell^44^,^45^,^46^,^47^,^48^,^49^ and the evolution of certain alleles in the Duffy blood group system which code for altered blood cells that do not allow *Plasmodium vivax* to enter the cell^50^,^51^.

The difficulty of finding incontrovertible evidence to support genetic hypotheses has resulted in a variety of alternative perspectives which de-emphasize the genetic hypotheses as missing key points^2^,^52^. These environmental hypotheses focus instead on other factors of virgin soil epidemics that differentially affected indigenous populations, namely famine, overcrowding, warfare, psycho-social stress, social chaos, and, most importantly, the lack of childhood exposure to novel diseases. Numerous historical records document the severe impact of virgin soil epidemics and suggest that they are most lethal during first contacts when indigenous immune systems are mostly naïve (i.e., lacking immunity) to novel pathogens like measles, influenza, and smallpox^2^,^24^. Exposed to completely new classes of pathogens for the first time, individuals are overwhelmed by new infections because no antibodies have previously formed during childhood. In virgin soil epidemics, typically people of all ages get sick, often from multiple afflictions, which leads to a general lack of food and necessary childcare^52^,^53^,^54^,^55^. Some behavioral responses may also have worsened epidemics, such as not burying corpses or participating in burial rituals that promote disease transmission, as was recently observed in the 2014 Ebola outbreak in Africa^56^.

The genetic and environmental hypotheses are not mutually exclusive and together may help to explain the disease experiences of New World indigenous populations. Most authors acknowledge elements of both in their arguments. Indeed, genetic and environmental factors interact to form complex phenotypes, and adaptive and innate immune defenses and epidemiological profiles are no exception^57^,^58^,^59^,^60^. In brief, multiple mechanisms often contribute to compromised immunity and newly contacted native Americans may be disadvantaged by a combination of nutritional stress and homozygous and naïve immune systems. Regardless of the specifics behind underlying mechanisms driving virgin soil epidemics and massive depopulation, the overall result from the last 500 years of European colonization in the Americas and elsewhere has been a natural experiment of repeated external perturbations to large samples of indigenous populations.

Here we analyze the ethnohistoric record of Greater Amazonia over the last century including many first sustained peaceful contacts between native Amazonians and the outside world. While most of the above cited literature on virgin soil epidemics emphasizes the immense calamity of European contacts and associated indigenous depopulation, we document temporal trends in mortality rates from contact-related epidemics and focus on improvements in survivorship through time to make recommendations for future first contacts. While our data do not speak directly to genetic hypotheses, the rapid improvements in survivorship through time do implicate a strong role for environmental factors, including increased immunity and better health care and conditions.

## Results

### (a) Descriptive data

**Figure 1:**
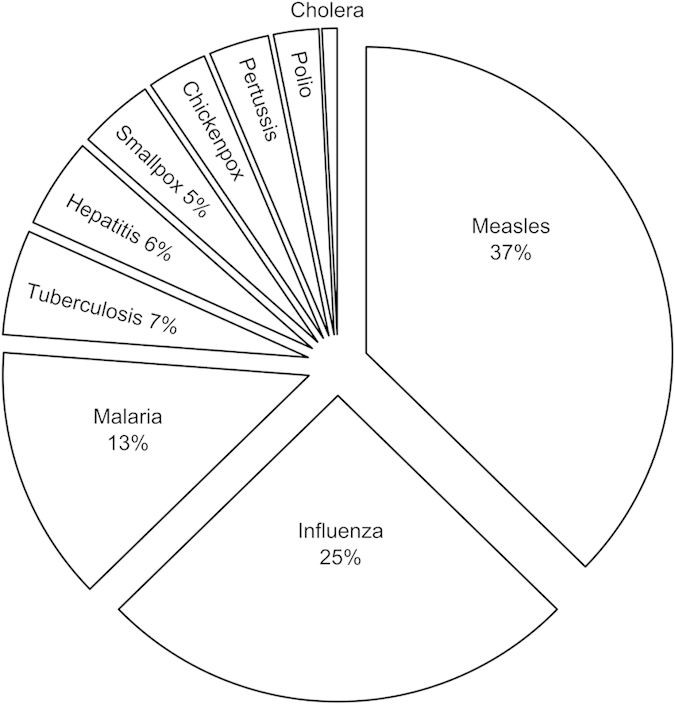
Pie chart of percentages of epidemics reported to have been caused by specific diseases. Multiple purported diseases are counted equally to give 126 total records.

Table 1 gives means and medians for the main variables in this study. The median epidemic in our sample occurred in 1965, afflicting indigenous populations of 180 people of which 18%, or 32 individuals, died within a year. The range of mortality rates across epidemics is immense, varying from less than 1% to 97%. We coded purported disease(s) involved in each epidemic (27 of 117 epidemics were missing a specific disease). Counting multiple diseases equally yields 126 total records (Fig. 1); of these the most common epidemics are 47 records of measles (37%), 32 of influenza (25%), and 17 of malaria (13%). Table 1 shows that the means and ranges of mortality rates are similar for epidemics of measles, influenza, and malaria. There are not enough data to compare mortality rates for the other diseases, and the lack of specific causes for many data points limits our ability to assess disease specific mortality comparisons. The estimated death count associated for each disease does appear to be approximately proportional to its occurrence because there are no statistically significant differences in mortality rates as a function of disease type when these are added as categorical variables to the linear mixed model.

**Figure 2:**
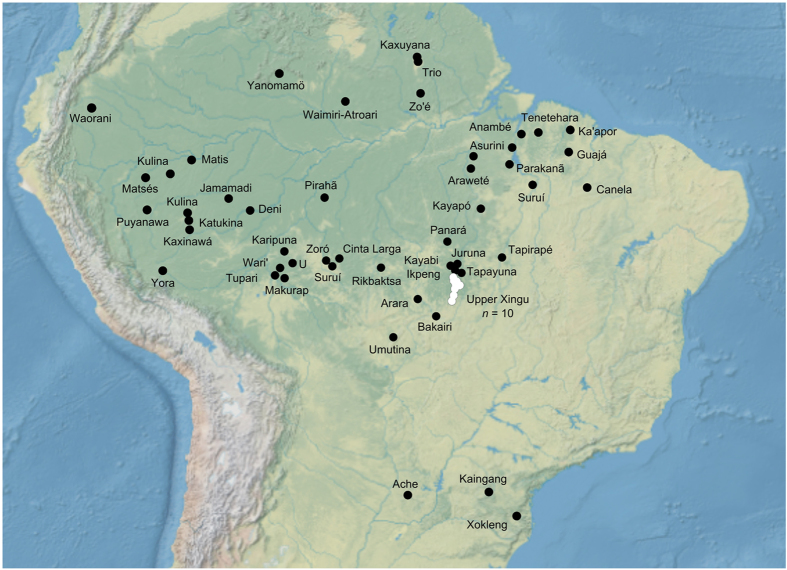
Locations of 59 indigenous societies in this study. White dots represent the hotspot of 10 societies conglomerated in the Upper Xingu region who suffered from epidemics of influenza in 1948 and measles in 1954 and 1965. Black dots represent other epidemics that appear to have only afflicted single societies. “U” is short for Uru-Eu-Wau-Wau. Map created in GenGIS software^77^.

We examined the spatial extent of particular epidemics by mapping the locations of outbreaks of the same disease and year. There are only 3 unequivocal examples of epidemics that spread to multiple societies, and these are all in same region. Figure 2 shows the hotspot of 10 societies in the Upper Xingu who suffered from epidemics of influenza in 1948 and measles in both 1954 and 1965^6^,^61^. All other epidemics in the dataset appear to have afflicted only single societies, or some diseases may have taken more than a year to spread to other locations that concealed the potential connection amongst different outbreaks.

### (b) Temporal trends in mortality rates

**Figure 3:**
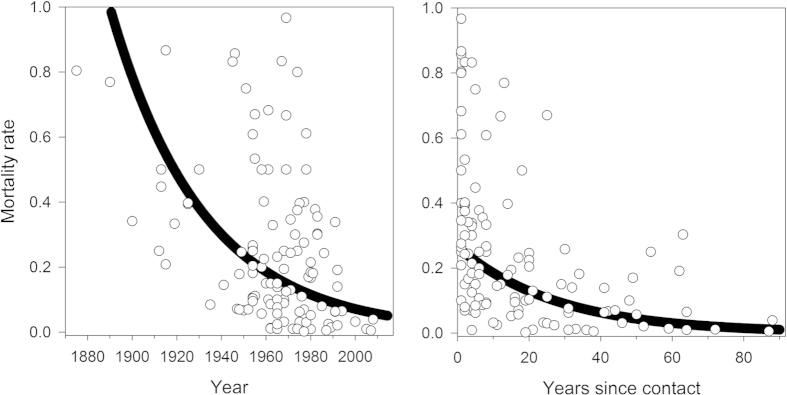
Mortality rates (fraction of population dying per year) from 117 epidemics as a function of absolute year (left) and years since contact (right). Both relationships are best fit with a negative exponential. High mortality outliers in the absolute year graph are from first contacts that occurred between 1950 and 1980.

Mortality rates measured as the fraction of population dying per year from epidemics (Fig. 3) decrease exponentially as a function of both absolute year (*y* = *ae*^−0.024*x*^, *n* = 117, *R*^2^ = 0.19) and time since contact (*y* = 0.26 *e*^−0.036*x*^, *n* = 117, *R*^2^ = 0.19). The Kayapó are the only society with sufficient data to look at a longitudinal trend, albeit from various subgroups that made sustained peaceful contact at different times^6^,^61^,^62^. The epidemics afflicting the Kayapó have mortality rates that also show an exponential decline with time since contact (*y* = 0.77 *e*^−0.123*x*^, *n* = 11, *R*^2^ = 0.62) but at a steeper rate than the cross-sectional fit above.

Table 2 shows results from a mixed effects model predicting mortality rates (natural logged) as a function of absolute year and years since contact. The parameter estimates for these variables from the mixed effects model are similar to those obtained in the bivariate relationships in Fig. 3. Moreover, there is no interaction effect between absolute year and years since contact. Therefore, we conclude that the time variables, both absolute time and relative time since contact, have mostly independent effects on mortality rates.

We show that the intensity of epidemics declines through time; further analysis shows how the frequency declines as well. Using the longitudinal record of those societies with multiple records of epidemics gives the time between successive outbreaks within a particular society. The inter-epidemic period averages around 7 years and positively correlates with years since contact (*r* = 0.50, *n* = 58, *p* < 0.001), a pattern also visible in Fig. 3 where most epidemics occur soon after contact. Hence, contact epidemics become less frequent within societies as time progresses.

## Discussion

Our results highlight the suffering that indigenous populations in Greater Amazonia experienced from an onslaught of infectious diseases introduced by colonization. An average mortality rate of 25% per year across epidemics speaks to the severity of these disease outbreaks. Similarly, our mixed effect model indicates that contacts occurring in the average year of 1963 led to mortality rates of 24% per year but are predicted to drop to 10% for a hypothetical contact in the year 2015. The 3 biggest killers in our dataset—measles, influenza, and malaria—combined for 75% of epidemics in our data. These diseases all likely arrived in the Americas with Europeans, but many of the indigenous populations were so isolated that they may have been spared the ravages of European-introduced diseases at the time of colonization. As Black’s^22^ and Cliff and Haggett’s^23^ work on island epidemics suggests, because of the small size and isolation of these populations, the epidemic patterns for most of the European-introduced diseases would tend to follow Bartlett’s^19^ Type III pattern—they would occur only rarely and spread quickly, infecting nearly everyone and causing high mortality, but they would likely be short-lived and would be followed by long periods of time where the disease was absent from the population. These time lags between epidemics would often be long enough that most or all of the people who survived and developed immunity to a disease during a previous epidemic would have completed their natural life span, leaving a population almost entirely without immunity to that disease. As a consequence, in many cases, each time a disease such as measles or influenza entered the population, the population level of immunity was so low that it would essentially have been a virgin soil outbreak, setting the stage for high morbidity and mortality.

This type of disease pattern does not require that affected indigenous populations experience direct exposure to non-indigenous groups. Instead the contact could be along a chain of neighboring populations, beginning with acculturated groups and extending eventually to isolated indigenous populations that interact with their nearest neighbors and contact with outsiders. As Fig. 2 shows, however, outbreaks are generally isolated to individual societies or regionally localized within remote areas as in the case of the 3 waves of epidemics in the Upper Xingu. That this hotspot of epidemics was recorded may in part be due to the fact that the Upper Xingu was particularly well studied and of high visibility. Alternatively, this region is also somewhat unusual in that first peaceful contacts made by the Villas-Bôas brothers and others occurred in many groups in a short time span and in several cases groups of people were moved into Xingu Park’s boundaries and into closer proximity with one another^6^,^61^,^63^. Regardless, it is likely that the generally localized pattern of outbreaks observed in Fig. 2 is a consequence of small populations and their relative isolation from each other, which makes the chances of widespread transmission between groups relatively rare before epidemics die out.

The health and contact situation has improved rapidly through time across Greater Amazonia, fortunately, and the few epidemics since the year 2000 all have mortality rates under 5%. Concurrently, survivorship also improves with time since contact and the best fit line predicts 1% mortality after 100 years post contact (Fig. 3). The secular trend of improving survivorship bodes well for future contacts. We did not, however, find an interaction effect between absolute time and relative time since contact which would have indicated that improvements with contact have progressively increased through time.

There are a number of limitations to our analysis. One of which is the sparsity of data for latter years, namely only 4 epidemics since the year 2000 that include 2 outbreaks of hepatitis (likely type A) with case fatality rates that are known to be low. However, this result is unlikely a bias in reporting because more recent outbreaks should be better documented than in the past. If real, this result corroborates the improved health situation where high-mortality epidemics from measles and other severe diseases are (hopefully) no longer occurring. A decline in mortality over time from vaccine preventable diseases may be due to these diseases no longer being introduced with the same probability as they were in the past, since new visitors are now much more likely to be vaccinated. Our study cannot directly test genetic hypotheses to explain the apparent susceptibility of native Amazonians to devastating epidemics, but rapid improvements in survivorship through time do suggest a strong role for environmental factors of better immunity and health care and conditions. We cannot determine the relative contribution of these two important factors given the general lack of genetic and serologic evidence and the many limitations of any ethnographic accounts of healthcare. We do have the impression that healthcare is generally becoming more available to most indigenous Amazonians through time, with more access to distant hospitals, small clinics in more villages, and periodic vaccination campaigns by governments and others. However, efforts are still quite underfunded and meager in the many remote areas^11^.

One of us (KRH) was on site within weeks of first peaceful contact with Ache (Paraguay 1978), Yora Nahua (Peru 1986), and Mashco-Piro (3 women in Peru 1983), and in a Yomiwato Matsiguenga community (Peru 1986) when they were extremely isolated and suffering from new contact related epidemics even though intermittent contact had occurred for 25 years. From these personal experiences, the most important lesson learned was that mortality can be reduced to near zero levels if the contact team is prepared to provide around-the-clock medical treatment on site for a sustained period of time and complement it with food supplementation. A well-designed contact implies minimal rather than catastrophic mortality and can be quite safe, compared to the disastrous outcomes from accidental contacts. But safe contact events require a qualified team committed to staying on site for years or even decades. For example, foreign missionaries provided great care with the Yora for up to 6 months, but when they decided to take a furlough dozens of people died within a few weeks. A similar situation occurred with Catholic missionaries at an Ache community in 1975. Care had been provided for a year but when the missionaries took a vacation, many people died. In contrast, there have been several success stories such as when a band of Northern Ache was contacted in 1978 (only 1 contact-related death out of 25 people). Missionaries and anthropologists injected antibiotics when primary respiratory infections progressed to pneumonia and provided food to the sick. Likewise, the Puerto Barra Ache were contacted by a family of missionaries in 1979 who have provided continual care to this day with excellent outcomes (0 contact-related deaths out of 35 people).

A previous study^14^ of population dynamics of most of these same indigenous Amazonian societies shows rapid depopulation before and after contact but with fast population rebound within a decade of contact at which point annual population growth rates average nearly 4% because the surviving population is composed of mostly reproductive adults (i.e., most epidemics disproportionately kill infants and elders). Despite the catastrophic mortality of indigenous Amazonians over the last 500 years, surviving populations are remarkably resilient and remain demographically viable in large part because the disease situation ameliorates quickly as a function of time since contact. A population viability analysis of recently contacted populations suggests that even groups as small as 50 people have reasonable survival prospects^14^. Taken together these results have positive implications for the long-term survival of currently isolated populations, provided they are immediately attended to by modern health care professionals and are allowed to maintain access to protected habitats large enough to support their subsistence needs.

We are now in a position to largely remove most of the environmental factors that were responsible for many deaths and social chaos of past contacts. The Type III disease pattern also has the advantage that epidemics are relatively easy to contain. The ethnographic sources we consulted corroborate our personal experiences and are clear on a number of points. Forced migration, frightened refugees, bad hygiene, food shortage, and lack of medicine were recipes for disastrous contacts, whereas airlifting patients to hospitals, missionary assistance, and even meager health care and support saved many lives^6^,^24^,^61^,^62^,^64^,^65^,^66^,^67^,^68^,^69^,^70^,^71^,^72^,^73^,^74^. Provisioning food, shelter, security, antibiotics, vaccines, and antiviral drugs, as well as encouraging isolation of sick and exposed individuals are just some of the basic logical first steps to mostly eliminate deaths, confusion, and despair that characterized previous contacts. Unfortunately, many intermittent contacts today are made by loggers, miners, narcotraffickers and others without the best interests of isolated people in mind^6^,^10^,^12^. Some missionary contacts have led to many deaths (e.g., Zuruahã in 1978, Zo’é in 1982, Ayoreo in 2008), although not well documented and hence not covered in our dataset. It is clear that dedicated professionals are indispensable components of well-planned contact teams. They should be willing to provide long-term and continuous health care to recently contacted communities and be able to quickly mitigate against all potential threats^75^.

### Methodology

We compiled information on 117 epidemics that affected 59 different indigenous societies in Greater Amazonia (Fig. 2) and caused over 11,000 deaths between 1875 and 2008. Much information comes from Ricardo and Ricardo^13^ and the accompanying website of the *Instituto Socioambiental* (http://pib.socioambiental.org). The website includes ethnographic entries for all indigenous societies in Brazil along with news stories from the 1960s to the present that often feature disease outbreaks. These website entries and other original ethnographic sources (Supplementary Information) were searched for the following index items: “epidemics”, “disease”, and “contact”.

Each record in our data includes ethnolinguistic name, year of contact (defined as first sustained peaceful contact with outsiders), estimated population size before the reported epidemic, year of epidemic, number of deaths or fraction of population dying, specific disease(s) if available, and time interval over which the epidemic (or string of epidemics) occurred. With this information we calculated years since contact, years between successive epidemics within ethnolinguistic populations, and the mortality rate of the epidemic (defined as the fraction of the population dying from an epidemic per year). Common ethnohistoric accounts are of the form: “In 1965, 32 of 180 people died from measles”. Therefore, we assume a one-year time interval for most epidemics. Only when mortality was measured over multiple years does this adjustment go into the denominator of mortality rate. Unfortunately, the majority of records are silent on the age distribution of records; the few exceptions only serve to highlight variation from “most deaths were children” to “most deaths were elderly”.

Mortality rates decline exponentially with both absolute year and years since contact. Therefore, we first natural-log transformed mortality rate and fit it with a linear mixed effects model in *R* (*lme4* package^76^). Years since contact and absolute year (centered on mean year of epidemics of 1963) are covariates; ethnolinguistic name is included as a random intercept because each society has anywhere from 1 to 11 separate records with an average of 2.

## Additional Information

**How to cite this article**: Walker, R. S. *et al.* Mortality from contact-related epidemics among indigenous populations in Greater Amazonia. *Sci. Rep.* **5**, 14032; doi: 10.1038/srep14032 (2015).

## Supplementary Material

Supplementary Information

## Figures and Tables

**Table 1:** Summary data for the variables used in this study, from 59 societies sampled an average of twice each.

**Variable**	**Median**	**Mean**	**Range**
Year of contact	1948	1946	1874–1987
Year of epidemic	1965	1963	1875–2008
Years since contact	8	18	1–88
Years between epidemics	6	7	1–34
Population size	180	324	11–5,000
Time interval	1	1.7	1–13
Mortality rate	0.18	0.25	0.002–0.97
Mortality rate (measles)	0.18	0.24	0.002–0.83
Mortality rate (influenza)	0.20	0.28	0.03–0.97
Mortality rate (malaria)	0.19	0.27	0.005–0.74

Population size is an estimate from before the epidemic, time interval is the years over which mortality was measured, and mortality rate is the fraction of the population that dies from an epidemic per year and is estimated for all epidemics and for specific diseases where noted.

**Table 2:** Regression results from a mixed effects model predicting mortality rates (natural log of the fraction of population dying per year) from epidemics.

**Variable**	**Estimate**	**SE**	***p*-value**
(Intercept)	−1.422	0.148	<0.001
Years since contact	−0.031	0.006	0.001
Year of epidemic	−0.016	0.005	<0.001
Society (59 levels)	0.963	0.169	<0.001

Society name is entered as a random intercept.

Year of epidemic was first centered on the mean year in the sample (1963) so that the intercept estimates mortality rates during contacts in 1963, or 24% per year.
