# Supplementary material for: Mortality from contact-related epidemics among indigenous populations in Greater Amazonia
Source: Sci Rep. 2015 Sep 10;5:14032. doi: 10.1038/srep14032 (PMC4564847; doi:10.1038/srep14032)
Supplement: Supplementary Information [file srep14032-s1.doc]

Supplementary Information for:

Mortality from contact-related epidemics among indigenous populations in Greater Amazonia

1^st^ Author: Robert S. Walker *^1^

2^nd^ Author: Lisa Sattenspiel ^1^

3^rd^ Author: Kim R. Hill ^2^

*Communicating author ([walkerro@missouri.edu](mailto:walkerro@missouri.edu))

Affiliations:

^1^ Department of Anthropology, University of Missouri, Columbia MO, USA

^2^ School of Human Evolution and Social Change, Arizona State University, Tempe AZ, USA

| Society | Year | First Contact | Time Interval (years) | Mortality Fraction | Disease(s) | Source |
| --- | --- | --- | --- | --- | --- | --- |
| Anambé | 1875 | 1874 | 1 | 0.804 | Smallpox | ISA |
| Jamamadi | 1890 | 1877 | 1 | 0.769 | Measles | ISA |
| Tenetehara | 1900 | 1897 | 1 | 0.341 |  | Gomes 1977 |
| Umutina | 1912 | 1911 | 1 | 0.250 |  | ISA |
| Kaingang | 1913 | 1912 | 1 | 0.500 | Influenza | ISA |
| Puyanawa | 1913 | 1908 | 1 | 0.447 | Measles | ISA |
| Juruna | 1915 | 1914 | 1 | 0.867 |  | Hemming 2003 |
| Xokleng | 1915 | 1914 | 3 | 0.625 | Influenza | Urban eHRAF |
| Umutina | 1919 | 1911 | 1 | 0.333 | Measles | ISA |
| Kaxuyana | 1925 | 1923 | 2 | 0.788 | Measles | ISA |
| Umutina | 1925 | 1911 | 2 | 0.795 | Pertussis | ISA |
| Ka'apor | 1930 | 1929 | 1 | 0.500 | Respiratory infections | Balee 1994 |
| Tapirapé | 1935 | 1930 | 3 | 0.254 |  | Wagley 1977 |
| Tapirapé | 1941 | 1930 | 1 | 0.145 | Influenza | Wagley eHRAF |
| Kayabi | 1945 | 1941 | 1 | 0.832 | Measles | Hemming 2003 |
| Naruvoto | 1946 | 1945 | 1 | 0.857 | Influenza | ISA |
| Tapirapé | 1947 | 1930 | 7 | 0.500 | Malaria, influenza | Flowers eHRAF |
| Kalapalo | 1948 | 1946 | 2 | 0.139 | Influenza | Heckenberger 2000 |
| Nahukuá | 1948 | 1934 | 2 | 0.357 | Influenza | Heckenberger 2000 |
| Ka'apor | 1949 | 1929 | 1 | 0.246 | Measles | Balee 1994 |
| Trio | 1950 | 1908 | 5 | 0.334 | Chickenpox | ISA |
| Kaxinawá | 1951 | 1946 | 1 | 0.749 | Measles | ISA |
| Trio | 1952 | 1908 | 1 | 0.070 | Influenza | ISA |
| Asurini Tocantins | 1954 | 1953 | 1 | 0.263 | Influenza | ISA |
| Aweti | 1954 | 1924 | 1 | 0.258 | Measles | ISA |
| Kalapalo | 1954 | 1946 | 1 | 0.267 | Measles | Heckenberger 2000 |
| Kamayurá | 1954 | 1946 | 1 | 0.608 | Measles | ISA |
| Kuikuro | 1954 | 1934 | 1 | 0.103 | Measles | Heckenberger 2000 |
| Nahukuá | 1954 | 1934 | 1 | 0.205 | Measles | Heckenberger 2000 |
| Naruvoto | 1954 | 1920 | 1 | 0.182 | Measles | ISA |
| Trumai | 1954 | 1938 | 1 | 0.095 | Measles | Heckenberger 2000 |
| Waurá | 1954 | 1948 | 1 | 0.250 | Measles | Heckenberger 2000 |
| Yawalapiti | 1954 | 1948 | 1 | 0.107 | Measles | ISA |
| Asurini Tocantins | 1955 | 1953 | 1 | 0.533 | Influenza | ISA |
| Tapirapé | 1955 | 1930 | 1 | 0.111 | Measles | Shapiro eHRAF |
| Tupari | 1955 | 1930 | 1 | 0.670 | Measles | ISA |
| Kayapó | 1958 | 1957 | 1 | 0.500 |  | Verswijver 1992 |
| Kayapó | 1958 | 1952 | 1 | 0.200 | Measles | Verswijver 1992 |
| Trio | 1958 | 1908 | 1 | 0.057 | Influenza | ISA |
| Kayapó | 1959 | 1957 | 1 | 0.166 | Influenza | Verswijver 1992 |
| Kayapó | 1959 | 1939 | 1 | 0.225 | Measles, influenza | Hemming 2003 |
| Mehinako | 1960 | 1948 | 1 | 0.150 | Influenza, measles | ISA |
| Ikpeng | 1961 | 1960 | 1 | 0.500 |  | ISA |
| Suruí Aikewara | 1961 | 1960 | 1 | 0.683 | Influenza | ISA |
| Yanomamö | 1961 | 1960 | 1 | 0.100 | Malaria | Smole 1976 |
| Bakairi | 1962 | 1890 | 1 | 0.011 |  | Picchi 1994 |
| Ka'apor | 1962 | 1929 | 8 | 0.099 | Respiratory infections | Balee 1994 |
| Rikbaktsa | 1962 | 1957 | 5 | 0.750 | Influenza, chickenpox, smallpox | ISA |
| Suruí Aikewara | 1962 | 1960 | 1 | 0.150 | Smallpox | ISA |
| Wari' | 1962 | 1955 | 7 | 0.601 | Influenza, measles, mumps, TB | Conklin 2001 |
| Kayapó | 1963 | 1959 | 1 | 0.329 | Polio | Hemming 2003 |
| Aweti | 1965 | 1924 | 2 | 0.278 | Measles | Heckenberger 2000 |
| Canela | 1965 | 1936 | 1 | 0.150 | Smallpox, influenza | Hemming 2003 |
| Kalapalo | 1965 | 1946 | 2 | 0.200 | Measles | Heckenberger 2000 |
| Kuikuro | 1965 | 1934 | 2 | 0.153 | Measles | Heckenberger 2000 |
| Nahukuá | 1965 | 1934 | 2 | 0.020 | Measles | Heckenberger 2000 |
| Trumai | 1965 | 1938 | 2 | 0.048 | Measles | Heckenberger 2000 |
| Waurá | 1965 | 1948 | 2 | 0.114 | Measles | Heckenberger 2000 |
| Yawalapiti | 1965 | 1948 | 2 | 0.463 | Measles | Heckenberger 2000 |
| Kayapó | 1967 | 1965 | 1 | 0.833 | Measles | Verswijver 1992 |
| Ikpeng | 1968 | 1960 | 1 | 0.089 |  | ISA |
| Kayapó | 1968 | 1953 | 1 | 0.194 | Malaria | Verswijver 1992 |
| Pirahã | 1968 | 1920 | 1 | 0.100 | Measles | ISA |
| Waorani | 1968 | 1958 | 1 | 0.032 | Polio | Larrick et al. 1979 |
| Yanomamö | 1968 | 1960 | 2 | 0.187 | Measles | Smole 1976 |
| Yanomamö | 1968 | 1960 | 1 | 0.250 | Measles | Chagnon 1992 |
| Cinta Larga | 1969 | 1965 | 4 | 0.500 | Measles, tuberculosis, hepatitis, malaria | ISA |
| Kayapó | 1969 | 1968 | 1 | 0.967 | Influenza | Heckenberger 2000 |
| Kayapó | 1969 | 1957 | 1 | 0.667 |  | Verswijver 1992 |
| Tapayuna | 1969 | 1968 | 1 | 0.500 | Influenza | ISA |
| Asurini Xingu | 1971 | 1950 | 1 | 0.130 | Influenza, measles | ISA |
| Parakanã | 1971 | 1970 | 1 | 0.346 | Influenza, malaria | Wagley 1977 |
| Tapayuna | 1971 | 1968 | 1 | 0.244 | Influenza | ISA |
| Trio | 1972 | 1908 | 1 | 0.005 | Measles | Hemming 2003 |
| Suruí Paiter | 1973 | 1969 | 1 | 0.300 | Measles | ISA |
| Arara | 1974 | 1972 | 2 | 0.750 |  | Wagley 1977 |
| Guajá | 1974 | 1973 | 1 | 0.800 |  | Cormier 2003 |
| Kayapó | 1974 | 1953 | 1 | 0.010 | Measles | Verswijver 1992 |
| Pirahã | 1974 | 1920 | 1 | 0.250 | Measles | ISA |
| Ache | 1975 | 1970 | 5 | 0.311 | Respiratory infections | Hill and Hurtado 1996 |
| Ka'apor | 1975 | 1929 | 13 | 0.406 |  | Balee 1994 |
| Panará | 1975 | 1973 | 2 | 0.793 |  | Hemming 2003 |
| Yanomamö | 1976 | 1960 | 2 | 0.220 | Influenza, measles | Ramos 1995 |
| Araweté | 1977 | 1976 | 1 | 0.400 |  | ISA |
| Panará | 1977 | 1973 | 2 | 0.139 | Malaria, influenza | Hemming 2003 |
| Parakanã | 1977 | 1976 | 1 | 0.275 | Influenza, malaria | Hemming 2003 |
| Waorani | 1977 | 1958 | 1 | 0.002 | Measles | Larrick et al. 1979 |
| Kayapó | 1978 | 1953 | 1 | 0.030 | Influenza | Verswijver 1992 |
| Yanomamö | 1978 | 1960 | 1 | 0.500 | Measles | Ramos 1995 |
| Zoró | 1978 | 1977 | 1 | 0.611 | Tuberculosis, influenza, malaria | ISA |
| Deni | 1979 | 1930 | 1 | 0.170 | TB | ISA |
| Cinta Larga | 1980 | 1965 | 1 | 0.052 | Measles | ISA |
| Guajá | 1980 | 1976 | 4 | 0.736 | Malaria, influenza | ISA |
| Guajá | 1980 | 1976 | 1 | 0.214 |  | Cormier 2003 |
| Matis | 1980 | 1976 | 4 | 0.037 |  | ISA |
| Zoró | 1980 | 1977 | 3 | 0.500 | Malaria, hepatitis | ISA |
| Waimiri-Atroari | 1981 | 1975 | 1 | 0.181 | Measles | ISA |
| Matis | 1982 | 1976 | 1 | 0.378 | Influenza | ISA |
| Karipuna | 1983 | 1981 | 2 | 0.600 |  | ISA |
| Makurap | 1983 | 1920 | 1 | 0.303 | Influenza | ISA |
| Matis | 1983 | 1976 | 1 | 0.356 |  | ISA |
| Uru-eu-wau-wau | 1985 | 1983 | 1 | 0.080 | Measles | Hemming 2003 |
| Yora | 1986 | 1984 | 2 | 0.486 | Respiratory infections | Hill and Kaplan 1989 |
| Kulina | 1987 | 1900 | 1 | 0.005 | Malaria | ISA |
| Zoró | 1988 | 1977 | 1 | 0.025 | Pertussis, hepatitis | ISA |
| Deni | 1989 | 1930 | 1 | 0.014 | TB, malaria | ISA |
| Kulina Pano | 1991 | 1950 | 1 | 0.064 | Malaria, pertussis | ISA |
| Yanomamö | 1991 | 1960 | 4 | 0.250 |  | Chagnon 1992 |
| Zo'é | 1991 | 1987 | 1 | 0.338 | Influenza | ISA |
| Deni | 1992 | 1930 | 1 | 0.191 | Measles | ISA |
| Katukina Rio Bia | 1992 | 1940 | 1 | 0.021 | Malaria | ISA |
| Yanomamö | 1992 | 1960 | 1 | 0.140 |  | Chagnon 1992 |
| Deni | 1994 | 1930 | 2 | 0.130 | Malaria, TB, measles | ISA |
| Araweté | 2000 | 1976 | 1 | 0.032 | Chickenpox | ISA |
| Matsés | 2005 | 1969 | 1 | 0.009 | Hepatitis | ISA |
| Matsés | 2007 | 1969 | 1 | 0.005 | Hepatitis | ISA |
| Pirahã | 2008 | 1920 | 1 | 0.039 | Malaria | ISA |

Sources:

ISA refers to *Instituto Socioambiental* ([http://pib.socioambiental.org](http://pib.socioambiental.org/))

eHRAF refers to electronic Human Relations Area Files ([http://hraf.yale.edu](http://hraf.yale.edu/))

Balée W 1994. Footprints of the Forest: Ka’apor Ethnobotany—the Historical Ecology of Plant Utilization by an Amazonian People. New York: Columbia University Press.

Chagnon NA 1992. Yanomamo. Harcourt.

Conklin BA 1989. Images of health, illness and death among the Wari’ (Pakaas Novos) of Rondonia, Brazil, San Francisco: University of California, San Francisco, PhD thesis.

Cormier LA. (2003) Kinship With Monkeys. New York: Columbia University Press.

Heckenberger M 2000. Epidemias, índio bravos e brancos: contato cultural e etnogênese do Alto Xingu, in Franchetto, Bruna, and Michael Heckenberger. Os povos do Alto Xingu: história e cultura. Ed. UFRJ Univ. Federal, 77-110.

Hemming J 2003 Die If You Must: Brazilian Indians in the Twentieth Century. London: Pan Macmillan.

Hill KR, Kaplan H 1989. Population and dry-season subsistence strategies of the recently contacted Yora of Peru. National Geographic Research 5.3: 317-334.

Hill KR, Hurtado AM 1996. Ache Life History: The Ecology and Demography of a Foraging People. Aldine de Gruyter, New York.

Larrick J, Yost J, Kaplan J, King G, Mayhall J 1979. Patterns of health and disease among the Waorani Indians of Eastern Ecuador. Med Anthropol 3:147–189.

Ramos A 1995. Sanumá memories: Yanomami ethnography in times of crisis. Univ of Wisconsin.

Silva M 2009. Romance de Primas e Primos: Uma Etnografia do Parentesco Waimiri-Atroari, Manaus, Brazil: Valer Editora.

Smole WJ 1976. The Yanoama Indians: A Cultural Geography. Austin, TX: University of Texas Press.

Verswijver G 1992. The Club-Fighters of the Amazon: Warfare among the Kaiapo Indians of Central Brazil. Rijksuniversiteit Te Gent: Gent.

Wagley C 1977. Welcome of Tears: The Tapirapé Indians of Central Brazil. Waveland Press.
